# Genome-Wide Identification and Evolutionary Analysis of *NBS-LRR* Genes From *Dioscorea rotundata*

**DOI:** 10.3389/fgene.2020.00484

**Published:** 2020-05-07

**Authors:** Yan-Mei Zhang, Min Chen, Ling Sun, Yue Wang, Jianmei Yin, Jia Liu, Xiao-Qin Sun, Yue-Yu Hang

**Affiliations:** ^1^Institute of Botany, Jiangsu Province and Chinese Academy of Sciences, Nanjing, China; ^2^Institute of Industrial Crops, Jiangsu Academy of Agricultural Sciences, Nanjing, China

**Keywords:** *Dioscorea rotundata*, *NBS-LRR* genes, pathogen defense, *R* gene, genome-wide analysis

## Abstract

*Dioscorea rotundata* is an important food crop that is mainly cultivated in subtropical regions of the world. *D. rotundata* is frequently infected by various pathogens during its lifespan, which results in a substantial economic loss in terms of yield and quality. The disease resistance gene (*R* gene) profile of *D. rotundata* is largely unknown, which has greatly hampered molecular study of disease resistance in this species. *Nucleotide-binding site–leucine-rich repeat* (*NBS-LRR*) genes are the largest group of plant *R* genes, and they play important roles in plant defense responses to various pathogens. In this study, 167 *NBS-LRR* genes were identified from the *D. rotundata* genome. Subsequently, one gene was assigned to the *resistance to powdery mildew8* (*RPW8*)*-NBS-LRR* (*RNL*) subclass and the other 166 genes to the *coiled coil* (*CC*)*-NBS-LRR* (*CNL*) subclass. None of the *Toll/interleukin-1 receptor* (*TIR*)*-NBS-LRR* (*TNL*) genes were detected in the genome. Among them, 124 genes are located in 25 multigene clusters and 43 genes are singletons. Tandem duplication serves as the major force for the cluster arrangement of *NBS-LRR* genes. Segmental duplication was detected for 18 *NBS-LRR* genes, although no whole-genome duplication has been documented for the species. Phylogenetic analysis revealed that *D. rotundata NBS-LRR* genes share 15 ancestral lineages with *Arabidopsis thaliana* genes. The *NBS-LRR* gene number increased by more than a factor of 10 during *D. rotundata* evolution. A conservatively evolved ancestral lineage was identified from *D. rotundata*, which is orthologs to the *Arabidopsis RPM1* gene. Transcriptome analysis for four different tissues of *D. rotundata* revealed a low expression of most *NBS-LRR* genes, with the tuber and leaf displaying a relatively high *NBS-LRR* gene expression than the stem and flower. Overall, this study provides a complete set of *NBS-LRR* genes for *D. rotundata*, which may serve as a fundamental resource for mining functional *NBS-LRR* genes against various pathogens.

## Introduction

Yams (*Dioscorea* spp.) are important food crops in tropical and subtropical regions of the world, belonging to the *Dioscorea* genus in the family Dioscoreaceae of the order Dioscoreales (Salawu et al., 2014). Their starchy tubers have high nutritional content, containing carbohydrates, vitamin C, essential minerals, and dietary fiber (Muzac-Tucker et al., 1993). It was proposed that three yam crops, *D. alata*, *D. trifida*, and *D. rotundata*, were domesticated independently and widely cultivated in Asia, America, and Africa (Scarcelli et al., 2019). In West Africa, yams serve as essential food crops, ranking second after cassava (Salawu et al., 2014). However, the productivity of yams is threatened by various pests and microbial pathogens, including nematodes, fungi, bacteria, and viruses (Mignouna et al., 2001; Coyne et al., 2006; Oyelana et al., 2011). The diseases caused by these pathogens not only severely reduce production but also affect the quality of the edible tissues (Salawu et al., 2014). This collectively contributes to the economic loss of the farmers. In the past, several studies have tried to collect germlines resistant to various pathogens and to map the resistance loci (Amusa, 2001; Mignouna et al., 2001, 2002; Bhattacharjee et al., 2018). However, no functional disease resistance gene (*R* gene) has been cloned from yam crops so far.

Plant *R* genes are a group of genes that specifically function against invading pathogens. During the past 30 years, over 300 *R* genes have been cloned from many plant species (Kourelis and van der Hoorn, 2018). These *R* genes encode proteins (*R* proteins) with diverse domain structures (Kourelis and van der Hoorn, 2018). Among them, genes encoding nucleotide-binding site (NBS) and leucine-rich repeat (LRR) domains represent the largest class of known *R* genes; these genes are named *NBS-LRR* genes. The translated NBS-LRR proteins are intracellular receptors that recognize the presence of pathogens. *NBS-LRR* genes originated anciently during the evolution of green plants (Shao et al., 2019). Three subclasses diverged soon after the origin of this gene family. The characteristic N-terminal domains, including the Toll/interleukin-1 receptor-like (TIR), coiled coil (CC), and resistance to powdery mildew8 (RPW8) domains, were found in the three subclasses. Accordingly, they were named as *TIR-NBS-LRR* (*TNL*), *CC-NBS-LRR* (*CNL*), and *RPW8-NBS-LRR* (*RNL*) genes, respectively (Shao et al., 2016). TNL and CNL proteins usually function as sensors to detect pathogens. The binding of the LRR domain to pathogen effectors causes a conformational change of the TNL or CNL protein, which subsequently induces multimerization of the TIR or CC domain, resulting in immune system activation (Andersen et al., 2018; Kourelis and van der Hoorn, 2018). Alternatively, some TNL and CNL proteins may be activated by monitoring the state of specific host proteins. When the state of these host proteins is altered by pathogen effectors, such as by phosphorylation or degradation, the CNL and TNL proteins will perceive it and are activated in a similar way to when directly activated (Kourelis and van der Hoorn, 2018). The *RNL* subclass has two lineages, each named by a functional gene, namely *ADR1* and *NRG1* (Collier et al., 2011; Shao et al., 2016). Both ADR1 and NRG1 proteins function in immune signal transduction but not pathogen detection. Furthermore, NRG1 proteins were found to act specifically in TNL signal transduction (Peart et al., 2005; Qi et al., 2018; Castel et al., 2019; Wu et al., 2019).

Generally, plant genomes harbor from dozens to more than a thousand *NBS-LRR* genes (Gu et al., 2015; Li et al., 2016; Shao et al., 2019). The maintenance of such a large number of *R* genes reflects a consequence of the long-term arms race between the plant and pathogens. Genomic and evolutionary studies have provided insights into how functional *R* genes were generated and preserved during plant evolution. Collective studies revealed that a considerable number of *NBS-LRR* genes are clustered on the chromosome, which is a result of frequent tandem duplication events (Meyers et al., 2003; Shao et al., 2014). The clustering organization provides a unique opportunity for creating high-sequence diversity and generating functional *NBS-LRR* genes (Kuang et al., 2004; Wroblewski et al., 2007). Understanding genomic organization and evolutionary patterns has greatly promoted functional *R* gene identification and utilization in rice, soybean, and many other crops (Ashfield et al., 2012; Shao et al., 2014; Zhang et al., 2015). Therefore, elucidating the complete profile of *NBS-LRR* genes in a plant genome would be of great help for the mining and utilization of functional *R* genes.

*D. rotundata* (white Guinea yam) is the most popular yam species cultivated in the West and Central Africa. The publication of the *D. rotundata* genome provides a valuable resource for understanding the *R* gene profile in this important crop (Tamiru et al., 2017). Here, a systematic analysis was performed to understand the domain structure, chromosomal organization, evolutionary mechanism, duplication type, and expression pattern. The bioinformatic analysis of the *NBS-LRR* gene profile in this study provides a fundamental resource for further mining functional *R* genes in *D. rotundata* and understanding the evolutionary pattern of this gene family.

## Results

### Identification of *NBS-LRR* Genes From the *D. rotundata* Genome

A total of 167 *NBS-LRR* genes (Supplementary Table S1) were identified from the *D. rotundata* genome using previously described criteria (see details in Materials and Methods section), accounting for approximately 0.6% of the 26,198 annotated genes. To assign these *NBS-LRR* genes to different subclasses, the protein sequences of all identified *NBS-LRR* genes were subjected to BLASTp analysis against the well-defined *Arabidopsis thaliana* NBS-LRR proteins (Zhang et al., 2016). The results showed that 166 of the 167 *D. rotundata NBS-LRR* genes belong to the *CNL* subclass, whereas only one belongs to the *RNL* subclass. None of the *TNL* genes were detected in the *D. rotundata* genome, which is consistent with reports of other monocot genomes that all lack *TNL* genes (Shao et al., 2016; Xue et al., 2020). One gene (*Dr02646.1*) encoding an atypical TIR domain and an atypical NBS domain was detected in the *D. rotundata* genome. However, this gene should be assigned to the *XTNX* gene family, not the *NBS-LRR* gene family according to the criteria described in our previous study (Zhang et al., 2017).

**FIGURE 1:**
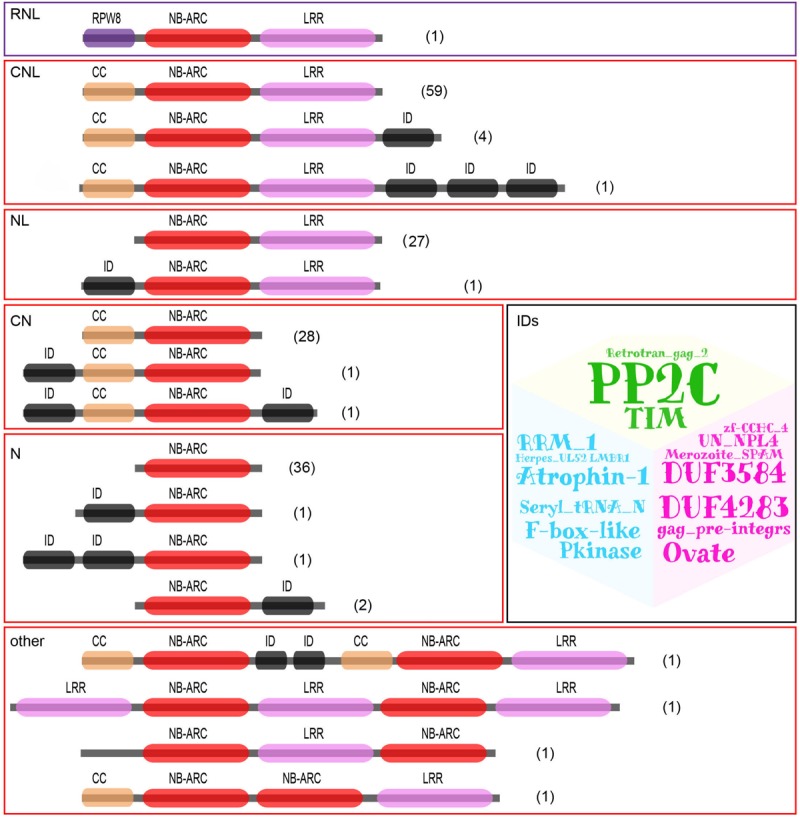
Identification and classification of *D. rotundata NBS-LRR* genes based on protein domain structure. 167 NBS-LRR genes were identified from the *D. rotundata* genome and classified into six groups with different domain compositions and arrangements. The identified integrated domains (IDs) are also listed as a word cloud.

Based on the domain combinations of the translated proteins, the 167 *NBS-LRR* genes were classified into six groups as illustrated in Figure 1. Two of the six groups contain intact *RNL* (one gene) or *CNL* (64 genes) genes, respectively. Genes in these two groups each encode an NBS domain, an LRR domain, and an N-terminal RPW8 or CC domain. Three other groups contain partial *CNL* genes, namely, *NL* (28 genes), *CN* (30 genes), and *N* (40 genes). These genes lack the N-terminal CC domain, the C-terminal LRR domain, or both, respectively. The remaining group of genes, namely, “others,” contains genes that also encode CNL proteins, yet have a complicated domain arrangement. Besides the characteristic domains regularly found in NBS-LRR proteins, 16 different integrated domains were detected to be encoded by 15 genes from the five *CNL* groups (Supplementary Table S1).

MEME analysis was performed on the amino acid sequence of NBS domain of *CNL* genes. The result showed that the highly conserved amino acid sequence of “GKTTLA,” “GLPL,” “DDVW,” and “TTR” at the four motifs P-loop, GLPL, Kinase-2 and RNBS-B are readily detected in *D. rotundata CNL* genes (Supplementary Table S2). Furthermore, the “DDVW” region in the Kinase-2 motif is conserved in both *CNL* and *RNL* genes as has been reported in other angiosperms (Shao et al., 2016).

### Chromosomal Distribution of *D. rotundata NBS-LRR* Genes

**FIGURE 2:**
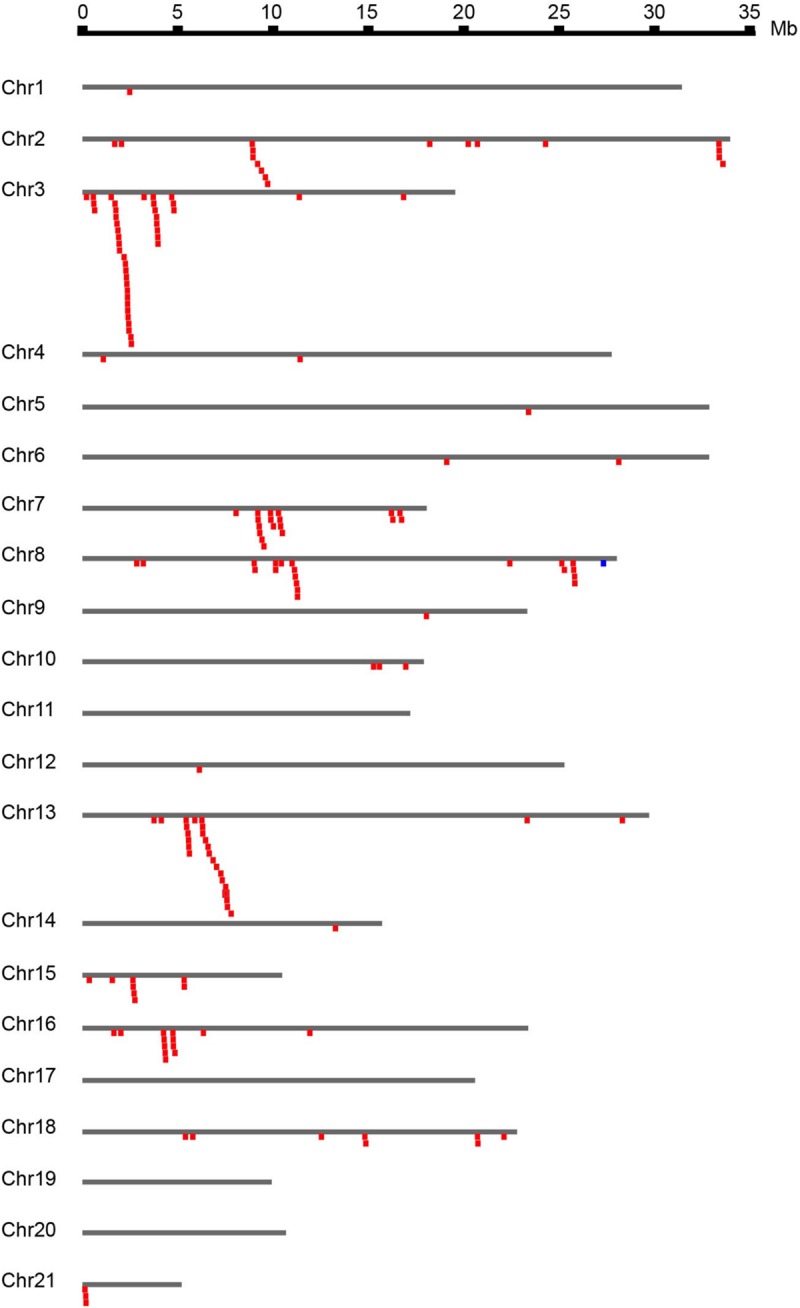
Chromosomal distribution of *D. rotundata NBS-LRR* genes. The 167 identified *NBS-LRR* genes are plotted against the *D. rotundata* chromosomes based on their physical locations retrieved from the GFF3 file. *NBS-LRR* genes within an interval of less than 250 kb were treated as a cluster (Ameline-Torregrosa et al., 2008).

The 167 identified *NBS-LRR* genes were plotted against the 21 *D. rotundata* chromosomes based on their physical locations retrieved from the GFF3 file. *NBS-LRR* genes within an interval of less than 250 kb were treated as a cluster (Ameline-Torregrosa et al., 2008). The result showed that *D. rotundata NBS-LRR* genes are unevenly distributed on 17 of the 21 chromosomes (Figure 2). More than ten *NBS-LRR* genes were detected on chromosomes 2, 3, 7, 8, 13, and 16, whereas only one *NBS-LRR* gene was detected on chromosomes 1, 5, 9, 12, and 14. No *NBS-LRR* genes were detected on chromosomes 11, 17, 19, and 20. No significant correlation was detected between the chromosomal length and the *NBS-LRR* gene number.

Based on the physical locations, the *NBS-LRR* genes on the 17 chromosomes were classified into 68 loci, including 43 singletons and 25 multigene clusters. The result demonstrated that 124 *NBS-LRR* genes are present in the 25 clusters. On average, there are five genes per cluster. Among the 25 defined clusters, the smallest ones only have two adjacent genes, including loci 27 and 28 on chromosome 7, loci 31, 32, and 36 on chromosome 8, locus 55 on chromosome 15, and loci 65 and 66 on chromosome 18. The largest cluster was locus 12 on chromosome 3, which has 23 *NBS-LRR* genes.

### Different Types of Gene Duplications Contributed to *NBS-LRR* Gene Expansion

**FIGURE 3:**
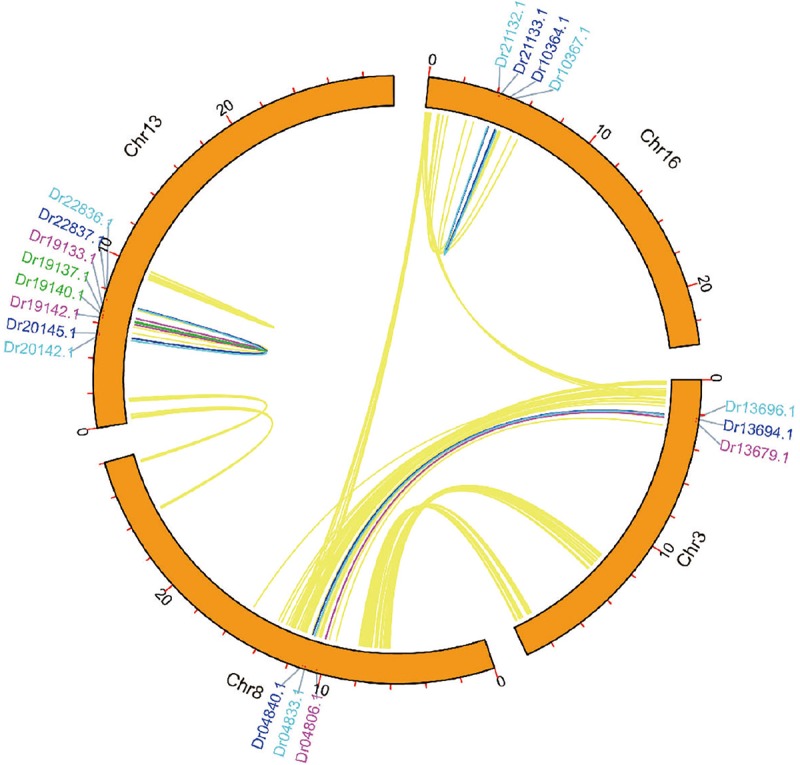
Syntenic relationship of the 18 segmental-duplicated *NBS-LRR* genes.

A large number of *NBS-LRR* genes were present in clusters, suggesting that tandem duplication plays an important role in the *D. rotundata NBS-LRR* gene expansion. Thus, the output of different duplication types was detected. The result showed that 108 of the 167 genes were duplicated through tandem duplications, 18 resulted from segmental duplications, and 41 were from ectopic or dispersed duplication. It is interesting that over 10% of the *NBS-LRR* genes originated from segmental duplications, whereas no whole genome duplications have been detected for the *D. rotundata* genome (Tamiru et al., 2017). Further analysis revealed that the 18 segmental duplicated genes are related to three segmental duplication events (Figure 3). One of them occurred between chromosomes 3 and 18, and resulted in duplication of three genes to form six. The remaining two events were intra-chromosomal small-scale inversions in chromosomes 13 and 16, resulting in the doubling of four and two ancestral *NBS-LRR* genes, respectively.

### Phylogenetic and Ka/Ks Analysis of *NBS-LRR* Genes From *D. rotundata*

**FIGURE 4:**
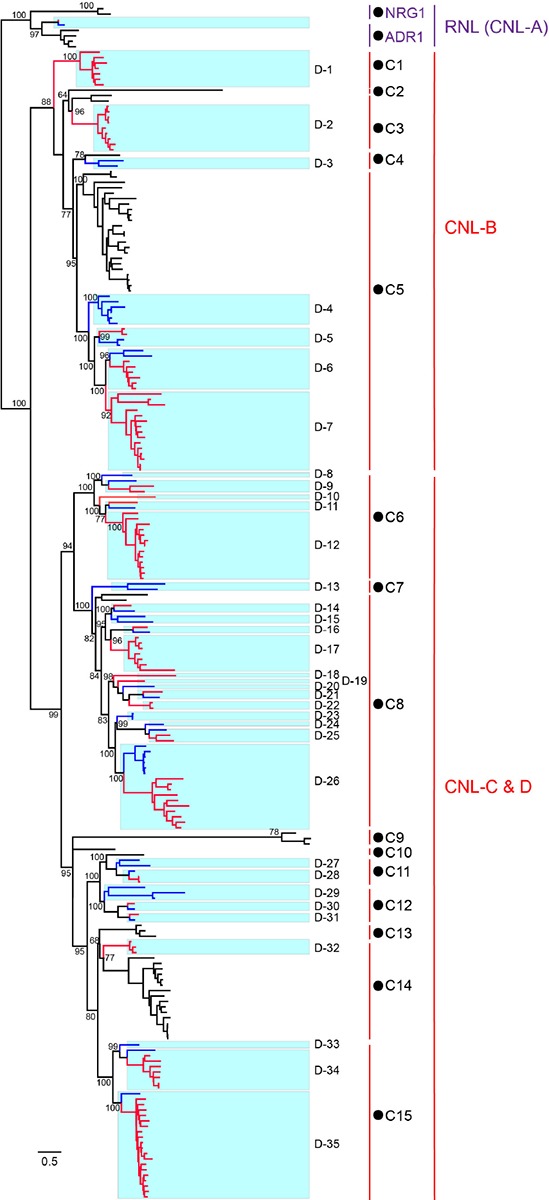
Phylogeny of *RNL* and *CNL* genes of *D. rotundata*, *T. zeylanicus*, and *A. thaliana*. The phylogeny was constructed based on the conserved NBS domain of *CNL* and *RNL* genes from *D. rotundata*, *T. zeylanicus* and *A. thaliana*. Branch support values obtained from a UFBoot2 test are labeled on basal nodes. Predicted ancestral lineages in the common ancestor of the three species (C1–C15) and ancestral lineages in the common ancestor of *D. rotundata* and *T. zeylanicus* (D1–D35) are indicated at the right of the phylogeny. The *CNL*-A, *CNL*-B, and *CNL*-C and D lineages are labeled according to Meyers et al. (2003).

To trace the evolutionary history of *D. rotundata NBS-LRR* genes, phylogenetic analysis was performed by incorporating *NBS-LRR* genes from the other Dioscoreaceae species *Trichopus zeylanicus* (Supplementary Table S3) and *CNL* and *RNL* genes from a dicot species *Arabidopsis thaliana* (Zhang et al., 2016). *TNL* genes of *A. thaliana* were not included in the analysis, because no *NBS-LRR* genes of this subclass were found in *D. rotundata*. The phylogenetic result (Figure 4 and Supplementary Figure S1) showed that *RNL* genes from the three species form an independent clade with a high support value, which corresponds to the *CNL*-A clade of *A. thaliana NBS-LRR* phylogeny constructed by Meyers et al. (2003). The topology supports the ancient divergence of the *RNL* and *CNL* subclasses documented by other studies (Shao et al., 2016, 2019). *RNL* genes from the three species further separated into two lineages, the ADR1 and NRG1 lineages. The *RNL* genes from *D. rotundata* and *T. zeylanicus* form a highly supported lineage with *Arabidopisis ADR1* genes, suggesting loss of NRG1 genes in the two species. This is in accordance with previous reported loss of *NRG1* genes in other monocot species (Shao et al., 2016). *CNL* genes from the three species also form two well-supported large clades (Figure 4). *D. rotundata* and *T. zeylanicus* genes in the first clade cluster with *A. thaliana CNL*-B clade genes, whereas *D. rotundata* and *T. zeylanicus* genes in the second clade cluster with *A. thaliana CNL*-C and D genes (Meyers et al., 2003).

Reconciling the *NBS-LRR* phylogeny revealed that *CNL* genes from the two Dioscoreaceae species and *A. thaliana* were derived from 15 ancestral lineages of the progenitor before the divergence of monocot and dicot plants (Figure 4). Among the 15 ancestral lineages, six were inherited by both *A. thaliana* and the Dioscoreaceae species (lineages 3, 4, 5, 8, 11, and 14). The lineage 5 has expanded a lot in both *A. thaliana* and the Dioscoreaceae species. In comparing, the lineage 3 and lineage 8 were only expanded in the Dioscoreaceae species. The lineage 11 was conservatively evolved in all of the three species, with only one to three genes in each genome. It is also worth noting that lineage 11 includes a functional *A. thaliana R* gene against *Pseudomonas syringae*, *RPM1*. Among the remaining nine lineages, four of them were inherited by *A. thaliana*, whereas five were inherited by the two Dioscoreaceae species (Figure 4).

In total, 11 of the 15 ancestral *CNL* lineages that emerged in the common ancestor of *A. thaliana* and the Dioscoreaceae species were inherited by the Dioscoreaceae species. These ancestral *CNL* lineages further diverged into 35 sub-lineages (D-1 to D-35) in the common ancestor of the two Dioscoreaceae species (Figure 4). Among them, eight sub-lineages (D1, D2, D6, D7, D12, D17, D26, and D35) had experienced considerable duplications in *D. rotundata*, resulting in a large number of descendant genes in the modern genome. Overall, the phylogenetic analysis revealed that the 11 ancestral *CNL* lineages that present in the common ancestor of monocots and dicots, have experienced step-wise expansion during *D. rotundata* evolution. This contributed to the expansion of the *NBS-LRR* gene number in *D. rotundata* to over ten times that of its ancestor.

The non-synonymous substitution to synonymous substitution (Ka/Ks) ratio is an informative value of positive selection. To detected whether some *NBS-LRR* genes are under positive selection, Ka/Ks analysis was performed on *D. rotundata NBS-LRR* genes from all aforementioned sub-families. The result showed that Ka/Ks for all but four gene pairs were less than one, indicating the majority of duplicated genes underwent purifying selection (Supplementary Table S4).

### Expression Profile of *NBS-LRR* Genes From *D. rotundata*

**FIGURE 5:**
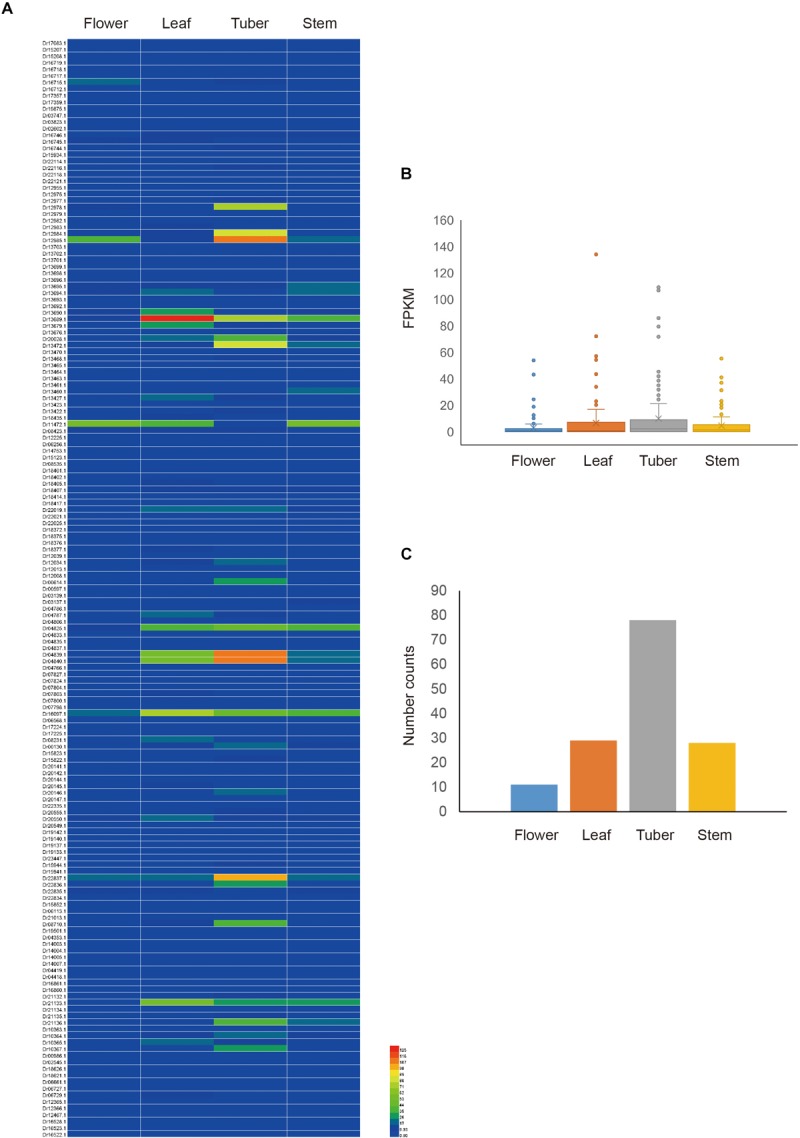
Expression pattern of *D. rotundata NBS-LRR* genes. **(A)** Heatmap of the expression of 167 *NBS-LRR* genes in four different plant tissues. **(B)** Average expression of 167 *NBS-LRR* genes in the four tissues. **(C)** Distribution of the top expression tissue for the 167 *NBS-LRR* genes.

To obtain the expression pattern of *NBS-LRR* genes in *D. rotundata*, the transcriptome data of four *D. rotundata* tissues from the public database were analyzed. The result showed that most *NBS-LRR* genes are not expressed or are only expressed at very low levels in all of the tissues studied (flower, leaf, tuber, and stem). However, the expression of some *NBS-LRR* genes could reach 100 fragments per kilobase million (FPKM; Figure 5 and Supplementary Table S5). Furthermore, the high expression of some *NBS-LRR* genes is often tissue specific. For example, among the five genes that were expressed at a level of more than 100 FPKM, four were highly expressed only in the tuber tissue.

The expression of all detected *NBS-LRR* genes was compared among the four tissues. The average expression value of the 167 genes is 2.9, 6.6, 9.9, and 4.6 FPKM in the flower, leaf, tuber, and stem, respectively. It is obvious that the tuber and leaf have higher average *R* gene expression levels than the stem and flower, although this difference is not statistically significant (Figure 5). The highest expression value of each gene was detected among the four tissues. The result showed that among the 146 genes that were expressed in at least one tissue, 78 genes show the highest expression value in the tuber, 29 in the leaf, 28 in the stem, and 11 in the flower (Figure 5). This mirrors the situation of the average expression level of all R genes among the four tissues. Overall, the expression analysis indicated that *NBS-LRR* genes in *D. rotundata* are expressed at a low level, with some genes showing high expression in specific tissues.

## Discussion

*NBS-LRR* genes are the largest group among all plant disease resistance genes, and they play vital roles in plant defense against various pathogens. Defining a complete set of *NBS-LRR* genes in a species is not only helpful for obtaining new insights into the evolution of this important gene family but also practical for the identification and utilization of functional *R* genes from the species and its close relatives (Meyers et al., 2003; Zhang et al., 2015). Genome-wide identification and evolutionary analysis has been performed in many angiosperms in the past 20 years (Bai et al., 2002; Meyers et al., 2003; Shao et al., 2016, 2019; Zhang et al., 2016; Neupane et al., 2018a). Several evolutionary features have been documented, including frequent tandem duplication for gene expansion, cluster organization on the chromosome, rapid species-specific gene loss, and duplication (Meyers et al., 2003; Gu et al., 2015; Shao et al., 2016; Die et al., 2018; Song et al., 2019). However, most of the previous studies have concentrated on dicots, especially the rosid lineage of the angiosperms. Only a few monocot species, mainly in the grass family, have been investigated. A recent study analyzed *NBS-LRR* genes in several genomes of the orchid family, which increased the catalog of analyzed lineages (Xue et al., 2020). In this study, this list was further expanded by analyzing *NBS-LRR* genes from *D. rotundata*, a species from an early diverged monocot lineage.

A comprehensive analysis of the 167 identified *NBS-LRR* genes clearly recovered previously documented evolutionary features of the *NBS-LRR* genes. The data revealed that 124 of the identified *NBS-LRR* genes are present within 25 clusters on the chromosomes. Furthermore, the cluster distribution of the *NBS-LRR* genes is consistent with their duplication mechanisms. The proportion of *NBS-LRR* genes that clustered reached 74%, which is higher than that of *A. thaliana* (Meyers et al., 2003). Segmental duplication of *NBS-LRR* genes is also frequently found in species that have recently experienced whole genome duplications (Shao et al., 2014). In this study, three segmental duplications involving 18 *NBS-LRR* genes were detected in *D. rotundata*, although no recent whole genome duplication has been recorded for this species (Tamiru et al., 2017). This result suggested that small-scale segmental duplications also play a role in *NBS-LRR* gene expansion. Identification and phylogenetic analysis of *RNL* genes from *D. rotundata* supports the stance that the NRG1 lineage has been lost in monocot lineages (Collier et al., 2011; Shao et al., 2016). Several recent studies have shown that many TNL proteins rely on NRG1 to transduce immune signals (Peart et al., 2005; Qi et al., 2018; Castel et al., 2019; Wu et al., 2019). The functional codependence of TNL and NRG1 was further strengthened by our observation that *NRG1* and *TNL* genes are co-absent in *D. rotundata*.

The arms race between *NBS-LRR* genes and plant pathogens drives rapid turnover of *NBS-LRR* profiles in a species. Therefore, conserved *NBS-LRR* lineages across different species are present at very low levels. The analysis of four legume species that diverged 54 million years ago revealed that over 94% (112 of 119) of ancestral *NBS-LRR* lineages experienced deletions or significant expansions during speciation. Meanwhile, only seven ancestral lineages were maintained in a conservative manner (Shao et al., 2014). In this study, the phylogenetic analysis of *NBS-LRR* genes from *D. rotundata* and *A. thaliana* revealed two ancestral *RNL* genes and 15 ancestral *CNL* genes between these two species that diverged over 100 million years ago. It is not surprising to see the loss of the NRG1 lineage and the preservation of only one copy of the ADR1 lineage *RNL* gene, because of their specific function in signal transduction rather than pathogen detection (Peart et al., 2005; Collier et al., 2011; Qi et al., 2018; Castel et al., 2019; Wu et al., 2019). Among the 15 ancestral *CNL* lineages, nine were lost in one of the two species. Only two of the remaining lineages were conservatively inherited by both species, whereas five lineages were expanded greatly in at least one species. It is interesting to find that the *A. thaliana RPM1* is located in one of the conservatively evolved ancestral lineages (lineage 11). *RPM1* has been proposed as an anciently originated *NBS-LRR* gene that defends against *P. syringae* (Mackey et al., 2002; Shao et al., 2016). RPM1 recognizes infection of the pathogen by monitoring a host protein, RIN4. Although maintenance of *RPM1* requires a high fitness cost (Tian et al., 2003), this gene has not been erased during long-term evolution, suggesting the importance of this anciently originated *R* gene and its functional mechanism. The finding of two *RPM1* orthologs in *D. rotundata* suggests that a function and mechanism similar to that of *RPM1* may have been adopted by monocot plants as well. It would be very interesting if this could be validated, because no *NBS-LRR* genes other than *RNL*s have been evidenced to maintain their function for such a long evolutionary time.

*NBS-LRR* genes are invaluable resources for mining functional *R* genes. A previous study in *D. alata* has tried to isolate *NBS-LRR* genes against Anthracnose by PCR (Saranya et al., 2016). However, PCR analysis can only be designed to amplify sequences encoding the conserved NBS domain without a reference genome. The full list of 167 *NBS-LRR* genes obtained in this study may serve as templates for mining full lengthen orthologous or homologous *NBS-LRR* genes from *D. alata* and other yam crops. The results from this study provide a fundamental resource for molecular breeding of *D. rotundata* and its relatives that have not yet been sequenced. The clustering of *NBS-LRR* genes on chromosomes has been associated with generating high sequence diversity and functional genes against various pathogens (Kuang et al., 2004; Wroblewski et al., 2007). In soybean, 54 million years of evolution has enabled one ancestral gene to be tandemly duplicated into more than ten offspring on chromosome 13, from which resistance has evolved to several different pathogens, including bacteria and different viruses (Ashfield et al., 2012; Shao et al., 2014; Nepal and Benson, 2015; Neupane et al., 2018b). Therefore, *NBS-LRR* clusters are excellent loci for mining functional *R* genes. In the present study, 25 clusters in *D. rotundata* aggregated 124 of the 167 *NBS-LRR* genes. Six chromosomes were found to have *NBS-LRR* clusters possessing more than five genes each (Figure 2). These clusters may serve as candidates for mining functional *R* genes against *D. rotundata* pathogens. However, the role of singleton *NBS-LRR* loci should not be neglected. It will be helpful to link the *NBS-LRR* locus identified in this study when genetic mapping is used for *R* gene discovery in *D. rotundata*.

In summary, the present study identified a complete set of 167 *NBS-LRR* genes from the *D. rotundata* genome. The genomic organization and evolutionary pattern were comprehensively revealed by integrating different analysis tools. These results may serve as a fundamental resource for the molecular breeding of *D. rotundata*.

## Materials and Methods

### Data Used in This Study

Genome sequence and annotation files of *D. rotundata* were downloaded from the Ensemble database^1^. The raw RNA-seq data (accession numbers: DRX040448, Flower; DRX040449, Leaf; DRX040450, Tuber; and DRX040451, Stem) generated by Tamiru et al. (2017) was downloaded from the National Center for Biotechnology Information (NCBI) sequence read archive (SRA) database. *Arabidopsis thaliana NBS-LRR* genes were retrieved from our previous study (Zhang et al., 2016).

### Identification of *NBS-LRR* Genes

BLAST and hidden Markov models search (HMMsearch) methods were used to identify *NBS-LRR* genes in the *D. rotundata* genome as described previously (Zhang et al., 2016). Briefly, the amino acid sequence of the NB-ARC domain (Pfam accession number: PF00931) was used as a query to search for NBS-LRR proteins using the BLASTp program of the NCBI BLAST software; the threshold expectation value was set to 1.0. Simultaneously, the protein sequences of *D. rotundata* were scanned by HMMsearch using the HMM profile of the NB-ARC domain as a query with an E-value setting of 1.0. Then, the results from the two methods were merged to produce the maximum number of *NBS-LRR* genes. In order to confirm the presence of the NBS domain, a round of HMMscan was performed for all the obtained hits against the Pfam-A database (*E*-value set to 0.0001). Genes without a conserved NBS domain were removed from the datasets. All of the non-redundant candidate sequences were compared with the NCBI Conserved Domains Database (CDD)^2^ and the MARCOIL server^3^ to further verify the CC, TIR (Pfam accession number: PF01582), RPW8 (Pfam accession number: PF05659), LRR, and other integrated domains.

MEME analysis (Bailey et al., 2009) was performed to discover conserved motifs in the NBS domain of the identified NBS-LRR genes. The number of displayed motifs was set to 20 with all other parameters default settings as described by Nepal et al. (2017).

### Distribution of *NBS-LRR* Genes in Different Chromosomes

To determine the distribution of the *NBS-LRR* genes on the chromosomes of the *D. rotundata* genome, the GFF3 annotation file was parsed to extract the genomic locations of the *NBS-LRR* genes. A sliding window analysis was performed with a window size of 250 kb to identify the number of genes that appeared in a cluster on a chromosome as described by Ameline-Torregrosa et al. (2008). If two successive annotated *NBS-LRR* genes were located within 250 kb on a chromosome, they were considered as clustered.

### Phylogenetic and Ka/Ks Analysis

Sequence alignment and phylogenetic analysis were performed as described by Xue et al. (2020). Briefly, amino acid sequences of the conserved NBS domain of the identified *NBS-LRR* genes were aligned using ClustalW with default options, and then manually corrected in MEGA 7.0 (Kumar et al., 2016). Too short or extremely divergent sequences were excluded from the analysis. Phylogenetic analysis was carried out with IQ-TREE using the maximum likelihood method (Nguyen et al., 2015) after selecting the best-fit model using ModelFinder (Kalyaanamoorthy et al., 2017). Branch support values were estimated using UFBoot2 tests (Minh et al., 2013). Reconcile the phylogeny was performed as described in our previous studies (Shao et al., 2014; Zhang et al., 2016) to reconstruction the ancestral state of the *NBS-LRR* genes.

The Ka and Ks were calculated for gene pairs within each NBS-LRR subfamilies. The nucleotide coding sequences (CDSs) of each subfamily were aligned by MEGA 7.0 (Kumar et al., 2016) and the values of Ka, Ks, and Ka/Ks were calculated by DnaSP (Librado and Rozas, 2009).

### Synteny and Gene Duplication Analysis

Pair-wise all-against-all BLAST was performed for the *D. rotundata* protein sequences. The obtained results and the GFF annotation file were then subjected to MCScanX for microsynteny detection and determination of the gene duplication type (Wang et al., 2012). Microsynteny relationships were displayed using TBtools^4^.

### Gene Expression Analysis

To analyze the expression of *D. rotundata NBS-LRR* genes, the RNA-seq data of various tissues were downloaded from GenBank and checked with FastQC software^5^ to avoid containing adapter or low-quality reads. Clean reads from each sample were mapped to the reference genome of *D. rotundata* using TopHat with default settings (Trapnell et al., 2012; Kim et al., 2013). The mapping results were subjected to Cufflinks to assemble transcripts in each sample and then merged into one cohesive set using Cuffmerge. The expression of each gene was evaluated using Cuffdiff (Trapnell et al., 2012). All analyses by cufflinks were performed with default settings. A gene with the FPKM value larger than 100 was recognized as a high expression gene in the analysis.

## Data Availability Statement

All datasets generated for this study are included in the article/Supplementary Material.

## Author Contributions

Y-MZ and Y-YH conceived and designed the project. Y-MZ obtained and analyzed the data and wrote the manuscript. MC, LS, YW, JY, JL, and X-QS participated in the data analysis and discussion. Y-YH revised the manuscript. All authors contributed to discussion of the results, reviewed the manuscript and approved the final article.

## Conflict of Interest

The authors declare that the research was conducted in the absence of any commercial or financial relationships that could be construed as a potential conflict of interest.

## Footnotes

1 ftp://ftp.ensemblgenomes.org/pub/plants/release-44/fasta/dioscorea_rotundata

2 http://www.ncbi.nlm.nih.gov/Structure/cdd/wrpsb.cgi

3 https://bcf.isb-sib.ch/webmarcoil/webmarcoilC1.html?tdsourcetag=s_pcqq_aiomsg

4 https://github.com/CJ-Chen/TBtools

5 http://www.bioinformatics.babraham.ac.uk/projects/fastqc/

## Supplementary Material

FIGURE S1 Detailed phylogeny of *NBS-LRR* genes from *Dioscorea rotundata*, *Trichopus zeylanicus* and *A. thaliana*.

TABLE S1 Detailed features of identified *NBS-LRR* genes.

TABLE S2 Discovered Motifs from NBS domain of *Dioscorea rotundata NBS-LRR* genes.

TABLE S3 *NBS-LRR* genes identified from *Trichopus zeylanicus*.

TABLE S4 Ka and Ks values of *Dioscorea rotundata NBS-LRR* pairs.

TABLE S5 Expression of *NBS-LRR* genes in the four *Dioscorea rotundata* tissues.
