# Supplementary figures and images for: Genome-Wide Identification and Evolutionary Analysis of NBS-LRR Genes From Dioscorea rotundata
Source: Front Genet. 2020 May 7;11:484. doi: 10.3389/fgene.2020.00484 (PMC7224235; doi:10.3389/fgene.2020.00484)

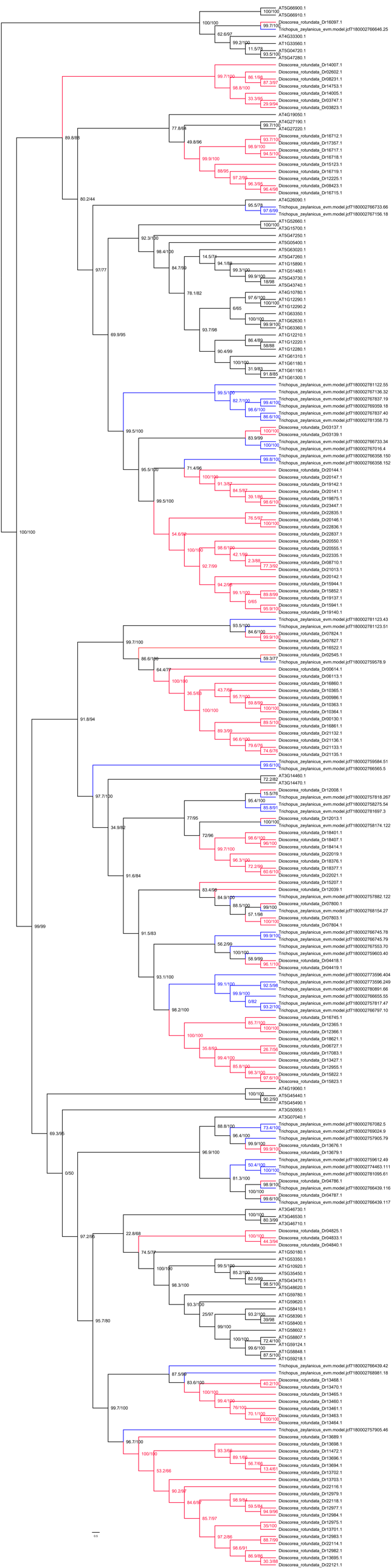

Supplement: FIGURE S1 — Detailed phylogeny of NBS-LRR genes from Dioscorea rotundata, Trichopus zeylanicus and A. thaliana. [file Data_Sheet_1.PDF]
