# Supplementary material for: Genome-Wide Identification and Evolutionary Analysis of NBS-LRR Genes From Dioscorea rotundata
Source: Front Genet. 2020 May 7;11:484. doi: 10.3389/fgene.2020.00484 (PMC7224235; doi:10.3389/fgene.2020.00484)
Supplement: TABLE S2 — Discovered Motifs from NBS domain of Dioscorea rotundata NBS-LRR genes. [file Table_2.DOCX]

**Table S2. Discovered Motifs from NBS domain of *Dioscorea rotundata NBS-LRR* genes.**

|  | **Logo** | **E-value** | **Sites** | **Width** |
| --- | --- | --- | --- | --- |
| 1.(p-loop) | 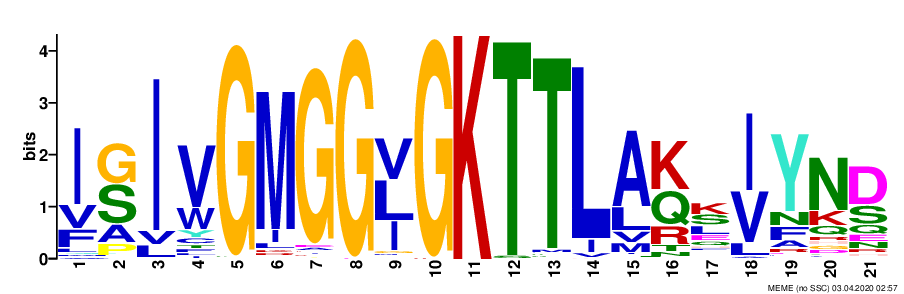 | 2.0e-1486 | 113 | 21 |
| 2. (GLPL) | 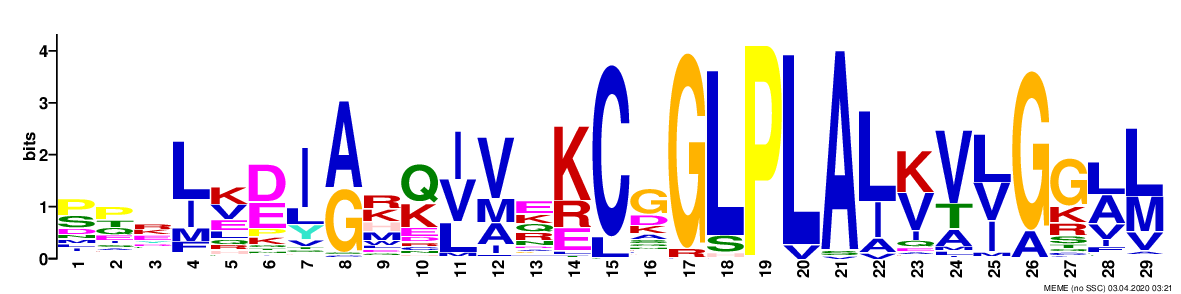 | 1.4e-1430 | 111 | 29 |
| 3. | 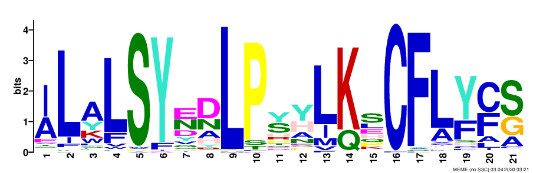 | 3.3e-901 | 78 | 21 |
| 4. (RNBS-B) | 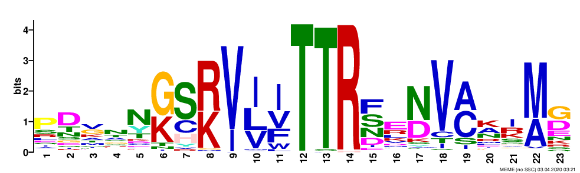 | 1.4e-968 | 100 | 23 |
| 5. (Kinase-2) | 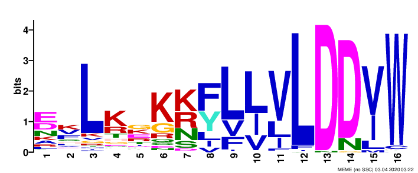 | 9.2e-723 | 116 | 16 |
| 6. | 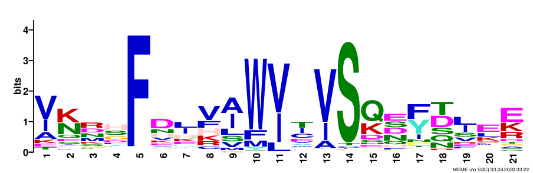 | 2.3e-682 | 113 | 21 |
| 7. (RNBS-C) | 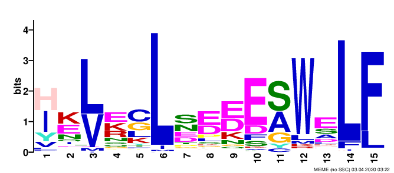 | 1.3e-562 | 117 | 15 |
| 8. | 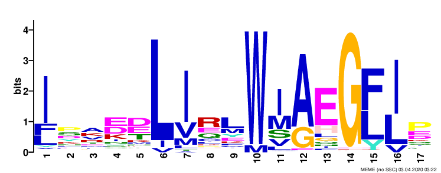 | 2.3e-509 | 92 | 17 |
| 9. | 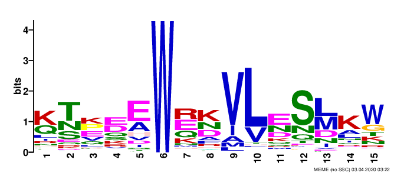 | 1.7e-333 | 100 | 15 |
| 10. (RNBS-D) | 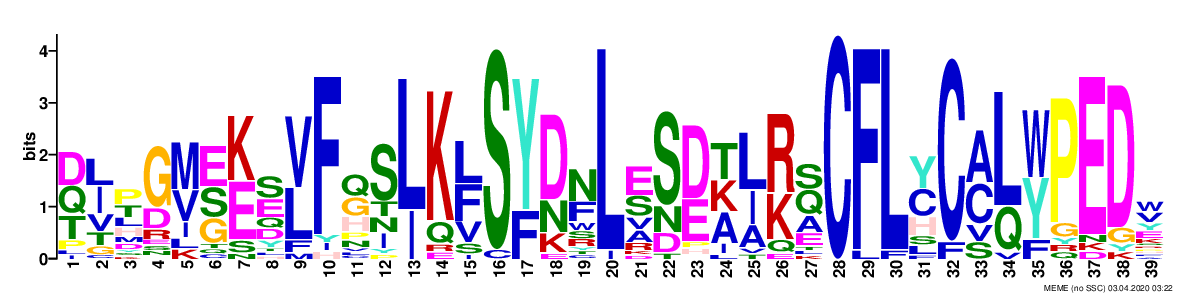 | 9.5e-343 | 22 | 39 |

|  | **Logo** | **E-value** | **Sites** | **Width** |
| --- | --- | --- | --- | --- |
| 11. | 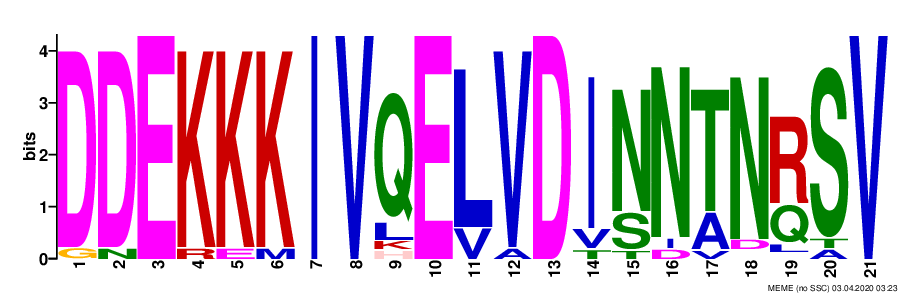 | 2.0e-216 | 18 | 21 |
| 12. | 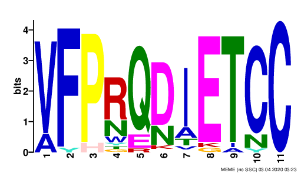 | 9.2e-145 | 23 | 11 |
| 13. | 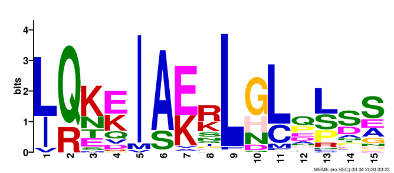 | 1.2e-127 | 37 | 15 |
| 14. | 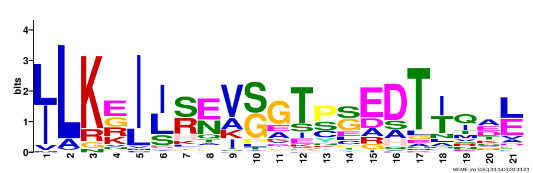 | 3.7e-184 | 45 | 21 |
| 15. | 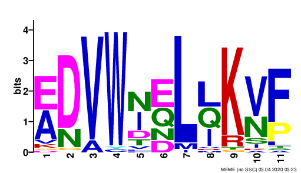 | 6.2e-126 | 36 | 11 |
| 16. | 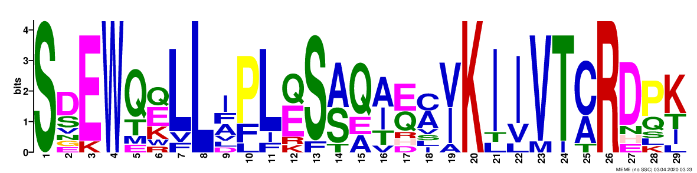 | 3.9e-082 | 10 | 29 |
| 17. | 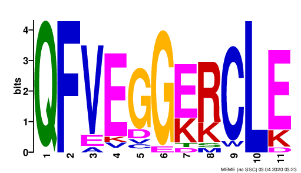 | 1.3e-076 | 18 | 11 |
| 18. | 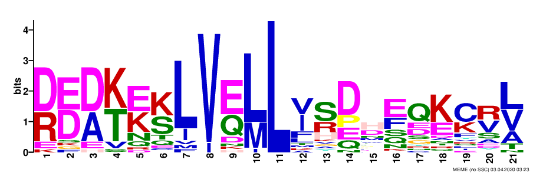 | 8.3e-067 | 23 | 21 |
| 19. | 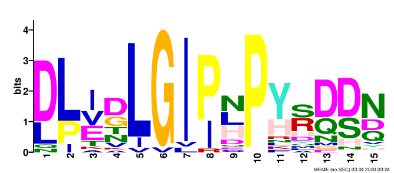 | 2.2e-075 | 21 | 15 |
| 20. | 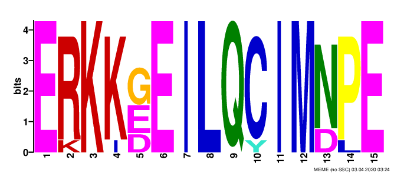 | 2.9e-065 | 9 | 15 |
